# Dicopper(II)-EDTA Chelate as a Bicephalic Receptor Model for a Synthetic Adenine Nucleoside

**DOI:** 10.3390/ph14050426

**Published:** 2021-05-02

**Authors:** María Eugenia García-Rubiño, Antonio Matilla-Hernández, Antonio Frontera, Luis Lezama, Juan Niclós-Gutiérrez, Duane Choquesillo-Lazarte

**Affiliations:** 1 Departamento Fisicoquímica, Facultad de Farmacia, Universidad de Granada, 18071 Granada, Spain; rubino@ugr.es; 2 Department of Inorganic Chemistry, Faculty of Pharmacy, University of Granada, 18071 Granada, Spain; amatilla@ugr.es (A.M.-H.); jniclos@ugr.es (J.N.-G.); 3 Departament de Química, Universitat de les Illes Balears, Crta. de Valldemossa km 7.5, 07122 Baleares, Spain; toni.frontera@uib.es; 4 Departamento de Química Orgánica e Inorgánica, Facultad de Ciencia y Tecnología, Universidad del País Vasco, UPV/EHU, 48940 Leioa, Spain; luis.lezama@ehu.es; 5 Laboratorio de Estudios Cristalográficos, IACT, CSIC-Universidad de Granada, Av. de las Palmeras 4, Armilla, 18100 Granada, Spain

**Keywords:** adenine, synthetic nucleoside, model receptor, copper, bicephalic chelate

## Abstract

In the extensive field of metal ions, their interactions with nucleic acids, and their constituents, the main aim of this work is to develop a metal chelate suitable to recognize two molecules of an adenine nucleoside. For this purpose, the dinuclear chelate Cu_2_ (µ-EDTA) (ethylenediaminetetraacetate(4-) ion (EDTA)) is chosen as a bicephalic receptor model for N9-(2-hydroxyethyl)adenine (9heade). A one-pot synthesis is reported to obtain the compound [Cu_2_(µ_2_-EDTA)(9heade)_2_(H_2_O)_4_]·3H_2_O, which has been characterized by single-crystal X-ray diffraction and various spectral, thermal, and magnetic methods. The complex unit is a centro-symmetric molecule, where each Cu (II) center is chelated by a half-EDTA, and is further surrounded by an N7-dentate 9heade nucleoside and two non-equivalent trans-O-aqua molecules. The metal chelate-nucleoside molecular recognition is referred to as an efficient cooperation between the Cu-N7(9heade) coordination bond and a (9heade)N6-H···O(carboxyl, EDTA) interligand interaction. Theoretical calculations are also made to account for the relevance of this interaction. The extreme weakness with which each water molecule binds to the metal center disturbs the thermal stability and the infrared (FT-IR) and electron spin resonance (ESR) spectra of the compound.

## 1. Introduction

Metal chelate interactions with nucleic acid bases (hereafter nucleobases) have aroused growing interest for decades in order to gain knowledge on the metalation processes of nucleosides, nucleotides, oligonucleotides, and nucleic acids. Metal binding patterns of nucleobases are now best understood in terms of molecular recognition (a universal point of view, always reciprocal, between the involved reactants) [1,2,3,4]. In this context, the versatility of adenine (Hade) as a ligand [2,4] is now understood to consist of the basicity (as proton affinity) of its N-atoms (N9 > N1 > N7 > N3 >> (exocyclic)N6), at the same time that the tautomerim(s) of the H(N9)-atom from its most stable tautomer, H(N9) ade. However, this does not seem applicable to its isomer 2-aminopurine [5]. When possible, the tautomer of Hade is used, as it favors the cooperating actions between metal-N(adenine) bonds and appropriate interligand H-bonding interactions. An example of the latter is the ternary copper(II) complex, derived from the tridentate N-benzyl-iminodiacetate(2-) chelator and the least stable tautomer H(N3)ade. In this molecular compound, Hade features the bridging µ-(N7,N9)ade coordination, by means of the cooperation of Cu-N9 and Cu-N7 bonds with N3-H···O(carboxylate) or N6-H···O(carboxylate) interligand interactions, respectively [6]. Copper(II) complexes have been recently used in medicinal chemistry and for molecular recognition [7,8,9].

On the other hand, the platination of the di(phospho-guanosine) dinucleotide (pGpG) with the kinetically inert cis-Pt (NH_3_)_2_^2+^ moiety in cis-[Pt(NH_3_)_2_[d(pGpG)]] revealed that the (anionic) terminal phosphate(2-) group efficiently overcomes the capability of the neutral exocyclic O6-guanine atom to act as an H-acceptor [10]. Today, the region-selectivity preference of the cis-Pt(NH_3_)_2_^2+^ moiety for adjacent guanine residues in repetitive pGpGpG… sequences (pG being 5′-phospho-guamosine) within a single-strand of nucleic acids is well understood. In contrast, the design anticancer platinum(II) compounds with a similar affinity for repetitive single-stranded pApApA… sequences (pA being 5′-phosphor-adenosine) represents a difficult chimerical question. In fact, the platination of N7-A by cis-Pt (NH_3_)_2_^2+^ moiety does not allow for its cooperation in a Pt(NH_3_)N-H···N6(ade) interaction, because of the hiperconjugation of the electronic lone-pair on N6 (Hade) with the purine moiety of an adenine residue. Therefore, in order to be able to carry out efficient cooperation between a metal-N7(adenine) bond of single-stranded (pA)_n_ with an interligand H-bond, some metal coligand(s) with appropriate H-acceptor atom(s) are needed. That is, instead of amino-N-H as H-donors, coordinated O atoms from carboxylate or alcoholic groups are needed.

**Scheme 1:**
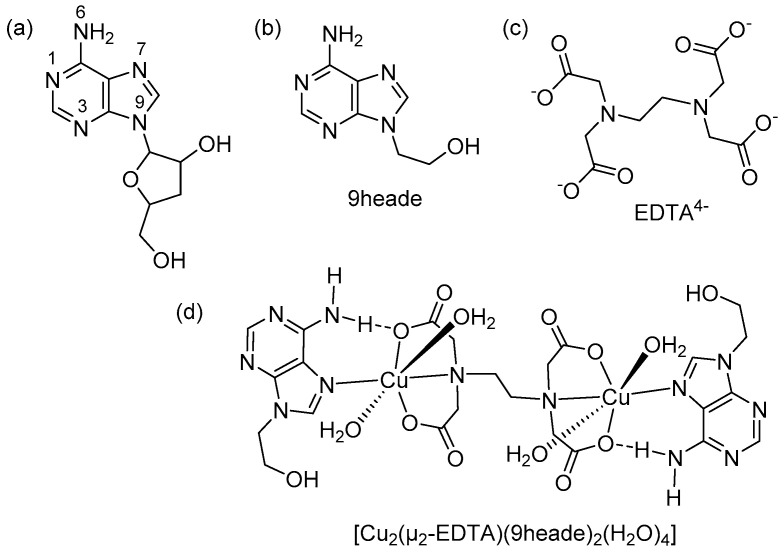
(**a**) Adenosine, (**b**) its synthetic acyclic analog 9heade, (**c**) the ethylenediaminetetraacetate(4-) ion (EDTA)^4–^′chelating ligands, and (**d**) the novel [Cu_2_(µ_2_-EDTA)(9heade)_2_(H_2_O)_4_] complex molecule.

Structural support for metal-purine nucleoside interactionz is surprisingly limited, most probably because of the difficulties in the crystalizing processes related to the opening–closure of the furanosyl ring and/or breaking of the N9-C1(sugar) bond. For these and other relevant reasons, metal interactions with synthetic purine nucleosides represent advantages and outline an extensive and promising field, as has been emphasized by Amo-Ochoa et al. [11]. In this context, significant advances have been made from crystal structures of a variety of ternary copper(II) complexes that bind acyclovir (acv) as coligand (see [12] and references therein). In these connections, we regard some metal complexes as receptors for the synthetic nucleoside N9-(2-hydroxyethyl)adenine (hereafter 9heade, Scheme 1), where the metal center is also linked to donor atoms of the coligands able to act as H-acceptors (O or Cl) atoms [13,14,15,16].

An actualized search of “9-(2-hydroxyethyl) adenine” in SciFinder affords only 44 closely related references, comprising papers or patents. This literature concerns its own synthesis [16,17], its use to obtain adefovir dipivoxyl [18], and other issues. The synthesis of well-defined DNA mimics brush polymers bearing adenine moieties [19]. The crystal structures of three polymorphs of 9heade have been known for a long time [20]. Interestingly, certain in vitro inhibitory effects of tumor cells to its 2′-phosphorilated form were reported [21], suggesting that such biological activities require the in vivo O-phosphorylation of its pseudo sugar -O(ol) H group. The structural knowledge of metal complexes with 9heade [13,14,15,16] strongly suggests that its preferred donor atom is N7 and that the N-basicity order for 9heade is N1 > N7 > N3 > >N6 (exocyclic) c. It is also clear that N7 is the preferred donor atom of 9heade, as supported by the crystal structures of various mixed-ligand complexes with Co(II) [15] Zn(II) [13,16], Cu(II) [13] or Cd(II) [14]. In addition, the bridging mode Cd_2_-µ-N1,N7(9heade) is documented for a Cd(II) coordination polymer [15]. Interestingly, in the crystal of {[Cu(gg)(9heade)(H_2_O)·Cu(gg)(μ_2_-9heade)]·8H_2_O}_n_ (gg = glycylglycinate(2-) anion), both the unidentate N7 and the bridging µ-N7,O(ol) are operative, also revealing that its O(ol) atom (mimicking the O2′-furanosyl adenosine atom) can contribute to supra-molecular recognition phenomena [13].

This work follows that reported by A. Domínguez-Martín et al. [9] on the molecular recognition between acyclovir (acv) and copper(II) chelates with N-(2-hydroxyethyl)-1,2-ethylenethediamine (hen), a flexible tridentate chelator that enables recognition between mononuclear Cu (hen) with one or two acv coligands. Our main aim is to design a binuclear Cu(II) chelate as a bicephalic receptor model for 9heade. With this aim, the Cu_2_(μ-EDTA) chelate (that crystallizes as {[Cu_2_(µ_4_-EDTA)(H_2_O)_2_]2H_2_O}_n_) [22] was chosen, because it is able to form binuclear ternary complexes with N-coligands, such as pyridines [23,24] or imidazole [25].

EDTA contains two potential tridentate iminodiacetate(2-) moieties (-N(CH_2_CO_2_^−^)_2_) linked by a reasonably flexible 1,2-ethylene spacer. Regarding this spacer flexibility, we premeditatedly excluded other related chelators possessing large and flexible spacers (such as n-polymethylene chains with n > 2), as well as other more or less rigid spacers (trans-1,4-bis (methylene)cyclohexane, p-phenylene or 1,4-xylylene). We also chose Cu(II) with metallic centers because it gives rather fast kinetic equilibriums with EDTA and with N-heterocyclic ligands, and also because it has been proven to form the above-mentioned binuclear compounds [23,24,25].

## 2. Results and Discussion

### 2.1. Comments Concerning the Synthesis

The synthesis of **1** follows the same strategy previously used to crystallize its imidazole (Him) analog, the polymer {[Cu_2_(μ_4_-EDTA)(Him)_2_(H_2_O)_2_]·2H_2_O}_n_ (OKEKUM in Cambridge Structural Database, hereafter CSD) [25]. Indeed, if Him or 9heade are not added, the procedure yields a potential receptor compound that crystallizes as the polymer {[Cu_2_(μ_4_-EDTA)(H_2_O)_2_]·2H_2_O}_n_ [22]. All these syntheses use a basic cooper(II) carbonate as a source of copper (II) and the EDTA chelating ligands in its acid form (H_4_EDTA). The four H^+^ ions from H_4_EDTA react with the carbonate and two hydroxide anions, yielding CO_2_ (as main byproduct) and three water molecules (from the solvent) according to the following acid­–base reaction:

CO_3_ = +2 OH^−^ + 4 H^+^ → CO_2_↑ + 3 H_2_O

Heating and stirring the reaction mixture, carbon dioxide (by-product) is easily removed. Hence, the overall process for the synthesis of **1** is written as follows:

Cu_2_(CO_3_)(OH)_2_ + H_4_EDTA + 2 9heade + 2 H_2_O → [Cu_2_(µ_2_-EDTA)(9heade)_2_(H_2_O)_4_]·3H_2_O↓ + CO_2_↑

Note that the final mother liquors contain 1 mmol of [Cu(H_2_O)_6_]^2+^ ions, 0.5 mmol of EDTA^4−^ chelator, and 1 mmol of 9heade in 100 mL of aqueous solution. Taking into account that the molar concentration of water is ca. 55.5 M, the binding of 9heade to Cu(II) to the Cu_2_(µ_2_-EDTA) chelate emphasizes the remarkable 9heade-Cu(II) ligand-to-metal affinity, which can be attributed to two non-excluding contributions. On the one hand, the bridging–chelating role of µ_2_-EDTA is very relevant. It is expected that each half of EDTA acts as a tridentate iminodiacetate chelator, thus occupying three of the four shortest and coplanar sites of copper(II) coordination [23,24,25]. This leaves the fourth nearest site available for the Cu-N7(9heade) metal binding, and two coordinated O-carboxylate groups as potential acceptors for a hydrogen bond, favoring an intended (9heade) N6-H···O (EDTA) interligand interaction. On the other hand, the affinity between N-heterocyclic ligands and second-half first-row divalent transition metal ions, M(II)(M = Fe-Zn), is well-known, reaching its strongest affinity when M = Cu. This well observed trend is rationalized under the general rule of “hard and soft” acids and bases of Pearson (HSAB), which states, “hard acids prefer bind with hard bases, and soft acids prefer bind soft bases”. All of these essential M(II) ions (M = Fe Zn) are unambiguously borderline Pearson’s acids and hence prefer joint borderline bases. The borderline basicity of Pearson is featured (among other donor atoms) in N-exocyclic amino groups for aromatic amines and N-heterocyclic donor atoms [26,27].

### 2.2. Molecular and Crystal Structure

Single crystal X-ray diffraction studies of molecular compounds, obtained by reactions of two (or more) reactants, are an essential source of information about the driving forces contributing to the molecular recognition between the chemicals involved in reactions. Indeed, crystallographic results can afford valuable structural information about molecular recognition, both at molecular and supramolecular levels. They clarify how molecules (with or without solvent ones) are formed and build stable crystalline networks [10]. The main aim of the present crystallographic study concerns to the way in which the binuclear chelate Cu_2_(μ-EDTA) joins up to two units of the synthetic adenine-nucleoside, 9heade.

**Figure 1:**
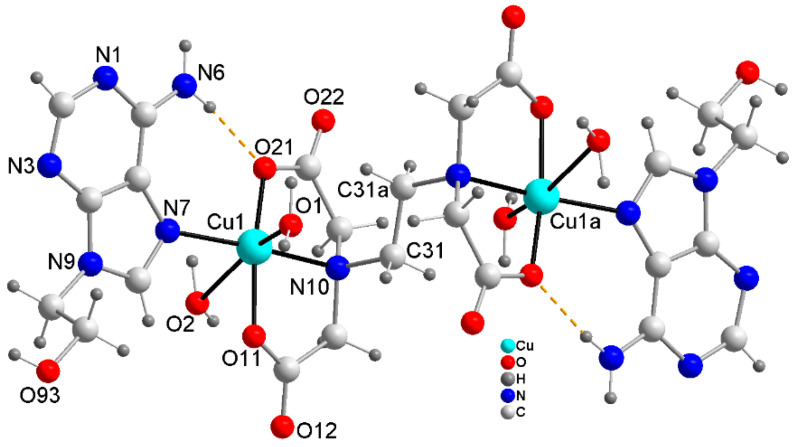
Centrosymmetric complex molecule [Cu_2_(µ-EDTA)(9heade)_2_(H_2_O)_4_] of compound **1** with the numbering notation used for some non-hydrogen atoms. Non-coordinated water is omitted for clarity. Symmetry code a (or ≠1 in supplementary material S4 and S5) = −x−1, −y, −z + 1 relates the two equivalent halves of this molecule.

The crystal of **1** consists of the complex molecule and three co-crystalized water molecules, according to the formula [Cu_2_(µ-EDTA)(9heade)_2_(H_2_O)_4_]·3H_2_O. Table 1 summarizes the coordination bond lengths and trans-angles (most relevant data of supplementary material). This trans-diaqua complex is a centrosymmetric molecule (Figure 1). Each half dinuclear complex has a tridentate N-substituted-iminodiacetate chelating group [-CH_2_-N(CH_2_-COO^−^)_2_] (hereafter IDA group) and the N7-unidentate 9heade nucleoside, with both occupying the four shortest (of about 2.0 Å) and coplanar coordination bonds with copper(II): Cu(1)-O(21), Cu(1)-O(11), Cu(1)-N(7) and Cu(1)-N(10) (Table 1). The Cu(II) surrounding is accomplished with two trans-asymmetrically distal aqua ligands, with rather unequal large Cu-O(aqua) bonds: Cu(1)-O(1) and Cu-O(2). The largest Cu(1)-O(2)-aqua distance (~2.82 Å) is rather close to the sum for the averaged atom radii, that is to the sum of so-called Van der Waals radii of Cu(1.40 Å) and O(1.52 Å), 2.92 Å [26].

**Scheme 2:**
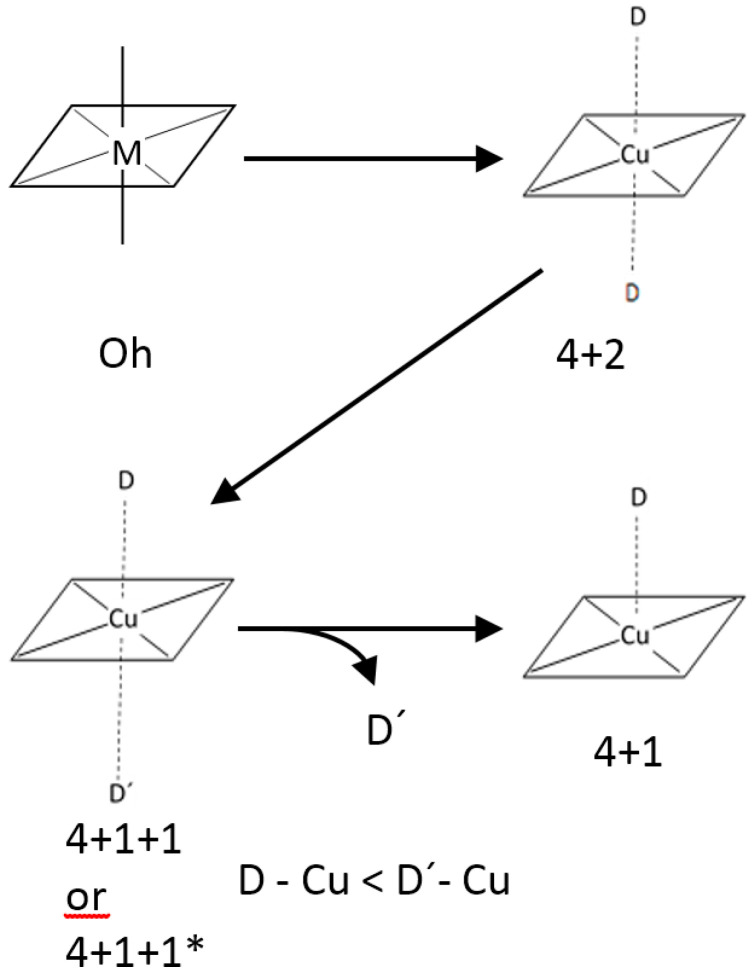
Distortions of a regular octahedron (Oh, for M ≠ Cu) toward other distorted six-coordination’s of Cu(II) (4 + 2, 4 + 1+1, 4 + 1 + 1*) to finally a distorted square-based Cu(II) coordination (4 + 1).

As a consequence of Jahn–Teller effect on the [Ar] 3d^9^ electronic configuration of Cu(II), all of their coordination polyhedra will exhibit a static (very rarely dynamic) distortion [28]. In compound **1,** such a distortion is essentially featured by the extremely trans-diaqua asymmetrical elongated coordination (Table 1). The Scheme 2 illustrates the progressive distortion from a regular octahedral coordination (Oh, with six close similar coordination bond distances) toward differently distorted six-coordinated Cu(II) coordinations, to finally reach a distorted five-coordination of Cu(II), type 4 + 1. The Cu(II) coordination polyhedron in compound 1 could be referred to as of type 4 + 1 + 1*, where * indicates that Cu1-O2 is a very weak interaction, close to a mere contact. This kind of polyhedron just precedes to a distorted square-based pyramid, type 4 + 1, in the elongations from a symmetric elongated coordination, type 4 + 2 (Scheme 2). In practice, such structural singularity [28] affects the thermal stability and spectral behavior of the compound reported here.

Therefore, ignoring the very weak Cu1···O2 (aqua) interaction, the copper(II) coordination in compound **1** is roughly described as a distorted square-based pyramidal coordination, type 4 + 1, with O1 (aqua) as a distal donor atom. In fact, the weak influence of the most distal O2 (aqua) donor moves the Cu(1) center ↑0.13 Å, from the mean basal plane (O11, O21, N7, N10) towards the least distal O1 (aqua) atom. The trans-basal N and O donors are roughly displaced (0.04 Å) towards the O1 atom, just up (N7,N10) or down (O11,O21) from this mean square plane.

**Figure 2:**
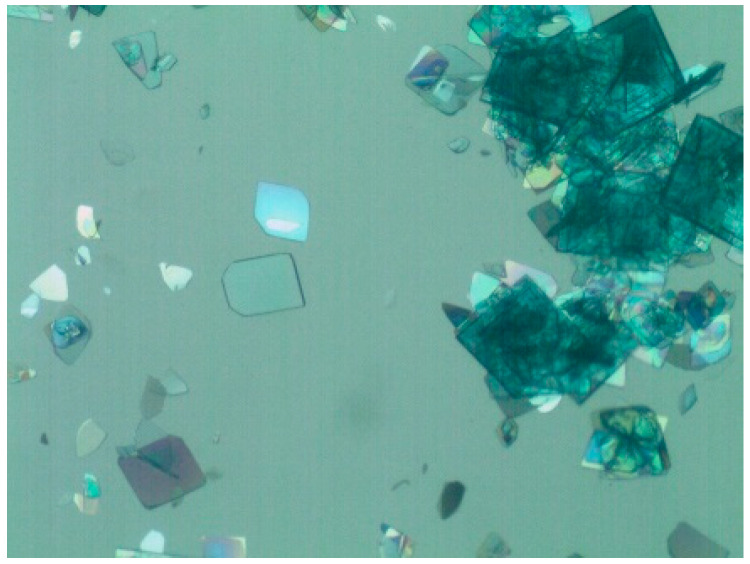
Crystals of **1** suitable for X-ray diffraction measurements. Photograph captured with polarized light, which allows for distinguishing the quality of the crystals.

As far as to the molecular recognition metal chelate-nucleoside is concerned, two facts are relevant. First, is the existence of cooperativity between each half of the complex molecule of **1** between the Cu1-N7 (9heade (2.03 (1) Å, see Table 1) and the interligand H-bond (9heade)N6-H6B···O21(EDTA) (see supplementary materials S5), where the O-carboxylate acceptor takes part of the Cu(II) chelation by the corresponding IDA group. Second, this molecular recognition pattern is the same for the two halves of the centrosymmetric complex molecule of compound **1** (Figure 2). The relevance of this pattern is underlined by the fact that working with a 100% of 9heade with respect to the amount used in the synthesis of **1** (see Section 3.1), the evaporation of the water (solvent) yields crystals of **1** (Figure 2) and colorless crystals of 9heade (both easily identified by its FT-IR spectrum).

**Figure 3:**
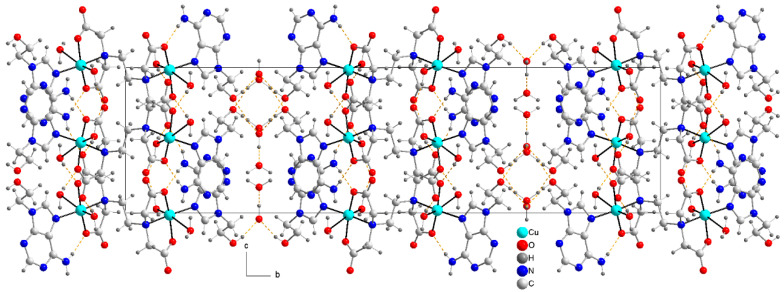
Crystal packing of compound **1**, showing H-bonding interactions that involve the (9heade)-O-H group and water molecules, connecting layers of complex molecules parallel to the ac crystal plane.

The crystal of compound **1** consists of a 3D H-bonding network (Figure 3), whereas π,π-stacking interactions between the adenine moieties are missing. H-donors are both hydrogen atoms of O-aqua ligands and O3-water molecules, H atoms from O4- and O5-water, and both N6-H bonds and the alcoholic O93-H bond of 9heade. H-acceptors are coordinated and non-coordinated O-EDTA atoms, the heterocyclic N1 and N3, and the alcoholic O93 atoms of 9heade, as well as all O atoms from water molecules. In this manner, H-bonded complex molecules fall in layers (parallel to the ac crystal plane), and they are connected by additional H-bonds also involving non-coordinated water molecules (Figure 3).

### 2.3. Computational Results

**Figure 4:**
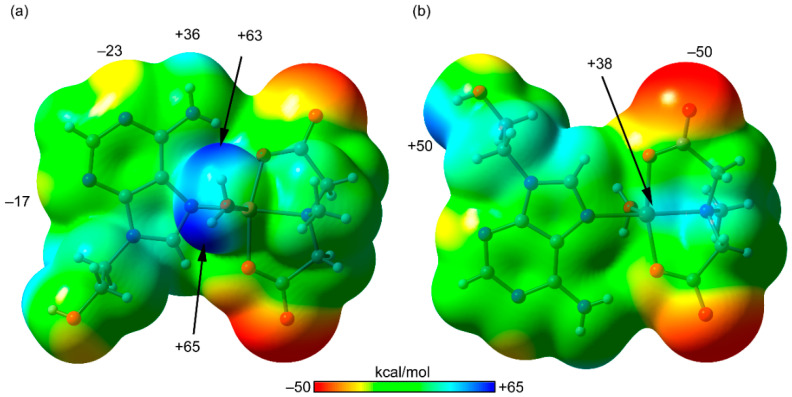
Molecular electrostatic potential (MEP) surfaces of the model of compound 1 using two different zenithal views: the coordinated water molecule pointing (**a**) up or (**b**) down using the 0.001 a.u. isosurface. The MEP values at selected points of the surface are indicated in kcal/mol.

**Figure 5:**
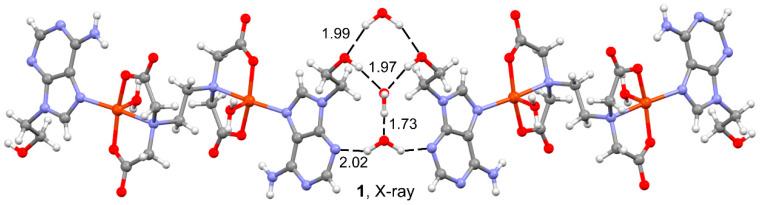
Partial view of the X-ray structure of compound **1** showing three co-crystallized water molecules connecting two [Cu_2_(µ-EDTA)(9heade)_2_(H_2_O)_4_] units. Distances are given in Å.

We have focused the theoretical analysis on the interesting assemblies and noncovalent interactions found in the solid-state structure of **1** that are relevant for the crystal packing. Firstly, we have computed the molecular electrostatic potential (MEP) surfaces of a theoretical model that is half of compound **1**; two different orientations are shown in Figure 4. It can be observed that the most positive region corresponds to the H-atoms of the coordinated water molecule (+65 kcal/mol) due to its coordination to the Cu(II) metal center that significantly enhances the acidity of these protons. Moreover, the hydroxyl H-atom of the pedant group of N^9^-hydroxyethyladenine also exhibits a large and positive MEP value (+50 kcal/mol). The exocyclic NH_2_ group presents a more moderate positive value of MEP (+36 kcal/mol, see Figure 4a), which is in fact similar to the MEP over the Cu metal center (+38 kcal/mol, see Figure 4b); therefore, both atoms are adequate to interact with electron rich atoms. Obviously, the most negative MEP values are located at the carboxylate groups (−50 kcal/mol). Therefore, from an electrostatic point of view, the H-bonds between the carboxylate and the coordinated water molecules are the most favored interactions, which is observed in the crystal packing of compound **1** (see supplementary materials S6). It is also worth mentioning that the alcohol group and the pyrimidinic N3-atoms of 9heade ligands establish H-bonding interactions with co-crystallized water molecules in good agreement with the MEP surface, which also shows large MEP values in these groups. It is worthy to highlight that two [Cu(μ_2_-EDTA)(9heade)_2_(H_2_O)_2_] units are connected in the solid state, by an interesting extensive network of H-bonds involving three co-crystalized water molecules that interconnect the hydroxyl group and N3-atom of two adjacent molecules (see Figure 5).

**Figure 6:**
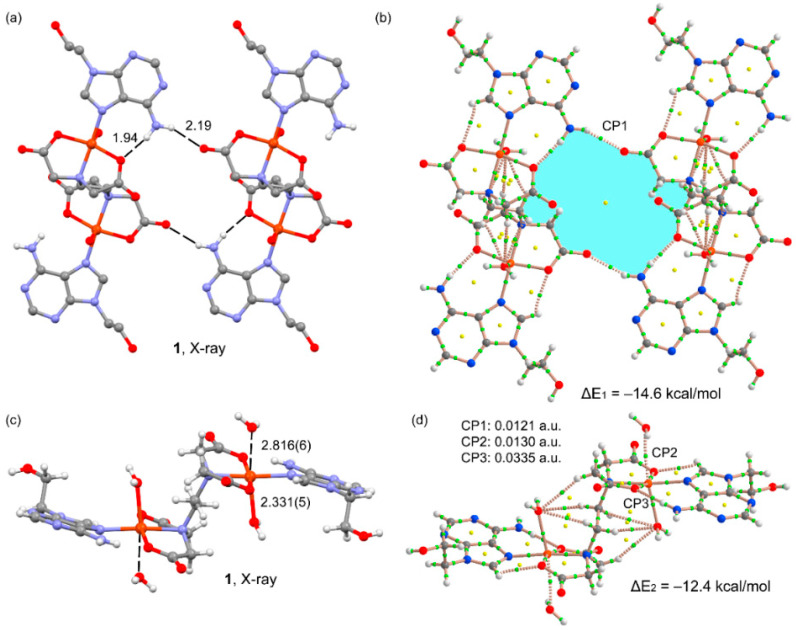
(**a**) Self-assembled dimer of **1** with indication of the intra- and inter-molecular H-bonds involving the amino group. (**b**,**d**) QTAIM distribution of bond and ring critical points (green and yellow spheres, respectively) and bond paths for two assemblies of compound **1**. (**c**) Assembly used to evaluate the Cu···OH_2_ interactions. The dimerization energies computed at the PBE0-D3/def2-TZVP are also indicated. All distances (**a**,**b**,**d**) are given in Å.

We analyzed two different assemblies, as highlighted in Figure 6. On the one hand, we studied the H-bonding interactions involving the exocyclic -NH_2_ group of the nucleobase as the H-bond donor and the coordinated carboxylate group as the acceptor (see Figure 6a,b). On the other hand, we also considered the nature of the Cu···OH_2_ interaction at the opposite side of the coordinated water molecule (see Figure 6c,d). The H-bond dimer shown in Figure 6a is formed by two symmetrically equivalent hydrogen bonds with a dimerization energy of ΔE_1_ = −14.6 kcal/mol, thus revealing that these H-bonds are (−7.3 kcal/mol each) in strong agreement with the anionic nature of the H-bond acceptor. The formation of these strong H-bonds likely prevents the formation of a coordination polymer, leaving Cu available to coordinate with a water molecule. We performed a quantum theory of “atoms-in-molecules” (QTAIM) analysis of the bond critical points (CPs) and bond paths on this self-assembled dimer (see Figure 6b). Each H-bond is characterized by a bond CP and bond path interconnecting the H-atoms to the O-atoms, thus confirming the contacts. Moreover, the AIM distribution also reveals the existence of an intermolecular NH···O H-bond involving the other N–H bond of the NH_2_ group.

The X-ray fragment used to analyze the Cu···OH_2_ interaction is given in Figure 6c, and it can be observed that the Cu···O distance is longer than the sum of the covalent radii of Cu and O (1.98 Å), but shorter than the sum of van der Waals radii (2.92 Å), thus indicative of a noncovalent interaction. The interaction energy obtained for the assembly where two Cu···OH_2_ contacts are established (represented in Figure 6d) is ΔE_2_ = −12.4 kcal/mol, thus each Cu···OH_2_ contact accounts for −6.2 kcal/mol. To further analyze the noncovalent nature of this contact, we included the magnitude of the charge density values at the bond CPs of the H-bond (CP1), the noncovalent Cu···O bond (CP2), and the coordination bond (CP3) in Figure 4. It can be observed that the values of density in CP1 and CP2 are very similar and indicative of a weak noncovalent interaction. In contrast, the value of ρ (r) at CP3 is much larger (more than twice) indicative of a coordination bond.

### 2.4. Thermal Stability

**Figure 7:**
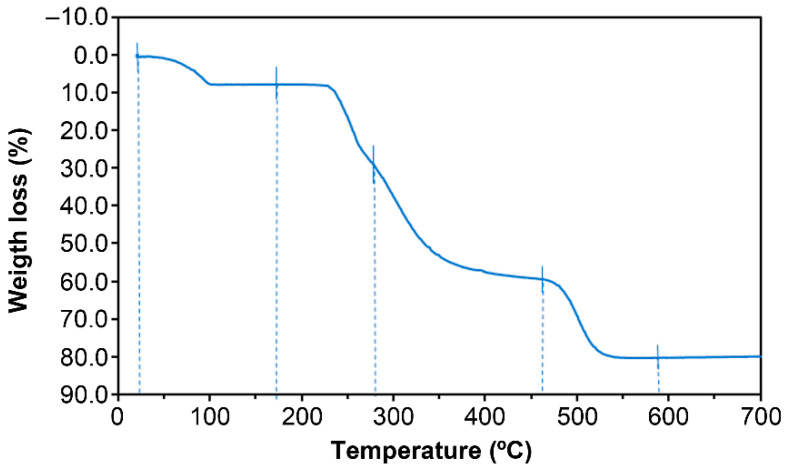
TGA of compound **1** under dry air flow.

More detailed information of this TGA study is supplied in the Supporting Material S.7. The weight loss during the TGA of a grounded sample (6.595 mg) of **1** under a dry-air flow is illustrated in Figure 7, which also shows four steps from r.t. to ~600 °C, giving a stable CuO final residue. The range of temperature for the first step (20–155 °C) was far too wide for the sample of **1** ([Cu_2_(µ-EDTA)(9heade)_2_(H_2_O)_4_]·3H_2_O, with formula weight 899.77) loses of both uncoordinated water and both distal aqua ligands (calculated value for 5 H_2_O (10.015%). The FT-IR spectra of the evolved gases during the first loss of weight revealed that only water was removed. However, the experimental water weigh loss (7.687%) in this step agreed was in agreement with a starting sample for formula [Cu_2_(µ-EDTA)(9heade)_2_(H_2_O)_3.6_] or Cu_2_(µ-EDTA)(9heade)_2_·3.6 H_2_O (formula weight 838.52). Indeed, the calculated value for the loss of 3.6 H_2_O from such a starting material (7.735%) was in excellent agreement with the above-referred experimental data. The remaining steps correspond to the burning of organics, with the production of ammonia and three N-oxides being essentially remarkable for the fifth step (270–600 °C). In order to assess the validity of such a starting composition, we can take into account that the experimental amount of CuO of the final residue (19.824%) is in good agreement with calculated value (18.973%, with an assumable deviation <1%). Note that the estimated formula for the starting material to the TGA experiment implies the complete absence of the three non-coordinated water molecules, and a partial loss of the more distal H_2_O(2) ligand when grinding the used sample.

### 2.5. Electron Spin Resonance (ESR) Spectra and Magnetic Properties

**Figure 8:**
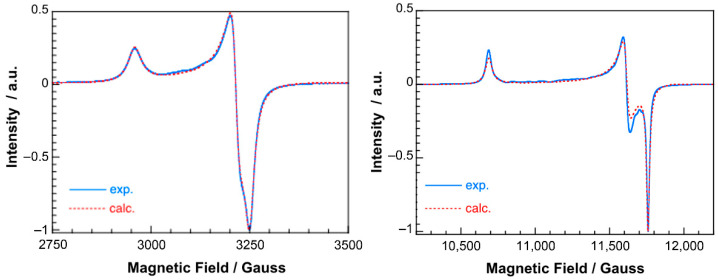
Experimental and simulated r.t. powder electron spin resonance (ESR) spectra of **1** recorded at (**left**) X-band and (**right**) Q-band.

The powder ESR spectra of this compound are rather sensitive to the grinding of its samples (supplementary materials S8). The best results show the characteristics of a rhombic g tensor at both X- and Q-bands (Figure 8). The spin Hamiltonian parameters have been determined by simulating the experimental spectra with a computer program working at the second order of the perturbation theory. The best-fit results are plotted as dashed lines in Figure 8. The main components of the g tensor are g_1_ = 2.270; g_2_ = 2.089; g_3_ = 2.064 (g_||_ = 2.270; g_⊥_ = 2.073; <g> = 2.141). These values are typical of Cu(II) ions in distorted square pyramidal environments with a d_x_^2^_−y_^2^ ground state. The signal is strongly exchange-narrowed, with a G parameter 3.7, which indicates that the local tetragonal axes of the molecules are only slightly misaligned [29]. Therefore, the calculated g values adequately reflect the characteristics of the environment of the Cu^2+^ ions in this compound.

**Figure 9:**
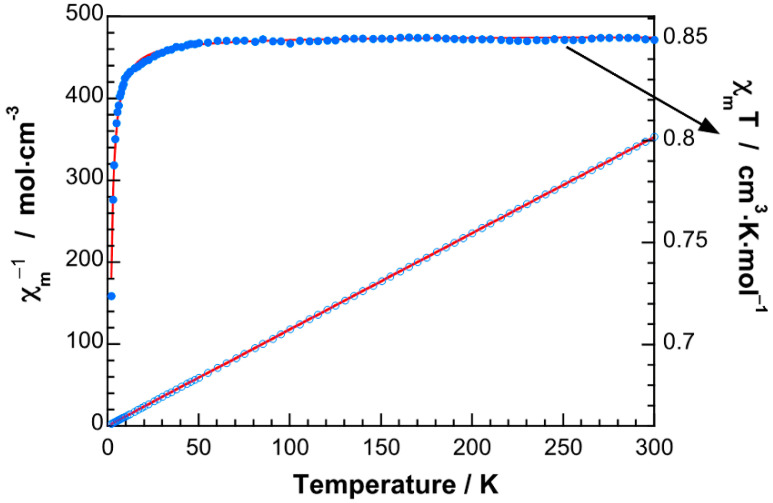
Magnetic properties of compound **1** (see text for the details of fit).

The magnetic properties in the form of *χ_m_*^−1^ and *χ_m_T* versus *T* plots (*χ_m_* being the magnetic molar susceptibility) are shown in Figure 9. The susceptibility data are well described by means of the Curie–Weiss expression in practically all of the recorded temperature ranges, being C_m_ = 0.85 cm^3^Kmol^−1^ and θ = −0.2 K. The C_m_ value is in good agreement with that expected for two magnetically non-interacting copper(II) ions with g = 2.141 (0.86 cm^3^Kmol^−1^). The magnetic effective moment remains practically constant from room temperature to 20 K, and then rapidly decreases at lower temperatures. This fact and the negative θ value in the Curie–Weiss expression are indicative of the predominance of very weak antiferromagnetic interactions in **1**. Moreover, the observed magnetic behavior corresponds to a system with prevailing short-range interactions. In this sense, a complete description of the observed behavior could be obtained using the Bleaney–Bowers equation for two coupled S = 1/2 ions [30]. The best fit of the experimental data to that in the equation (solid lines in Figure 9) is achieved for an exchange parameter of *J* = −0.35 cm^−1^ and g = 2.13, with *2J* being the singlet-triplet energy gap. The small *J*-value is a consequence of the disposition of the copper(II) ions in this complex, with their magnetic orbitals (mainly d_x_^2^−_y_^2^) in practically parallel planes and perpendicular to the exchange pathway.

### 2.6. Remarks on the Suitability of the Cu_2_(µ-EDTA) Chelate as Bicephalic Receptor for 9heade

The crystallographic results revealed that metal chelate and the synthetic nucleoside recognizes each other through efficient cooperation between the Cu-N7 (9heade) and a (9heade)N6-H···O (EDTA) H-bonding interaction. The bridging µ-EDTA role enables the dinuclear complex unit Cu_2_(µ_2_-EDTA) to act as a bicephalic receptor for 9heade. Note that for the use of a “receptor” concept (in this and other field’s related to biological and therapeutic chemistry), it is worth bearing in mind that the molecular recognition phenomenon is always reciprocal. Furthermore, molecular recognition far exceeds the field of chemistry.

**Figure 10:**
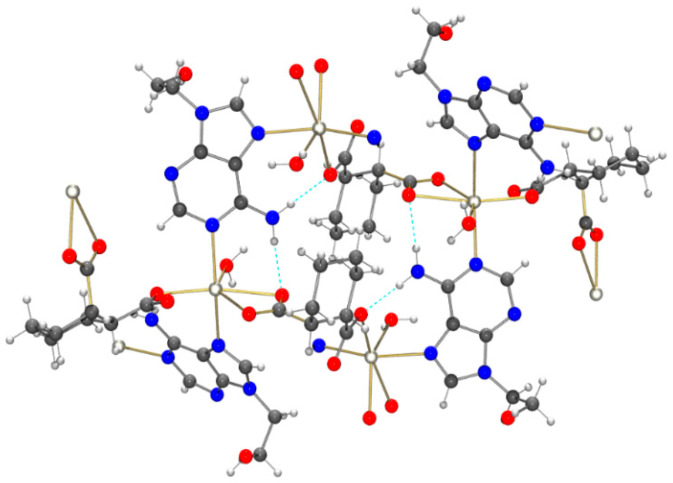
Fragment of the polymer {Cd(µ_2_-(cis-1,2-chdc) µ_2_-(N1,N7-9heade)(H_2_O)]·4H_2_O}_n_, showing (dashed cyan lines) the interligand (9heade)N6-H···O(carboxilato, cis-1,2- chdca) H-bonding interactions cooperating with the Cd-N1 and Cd-N7 coordination bonds. Non-coordinated water omitted for clarity.

**Figure 11:**
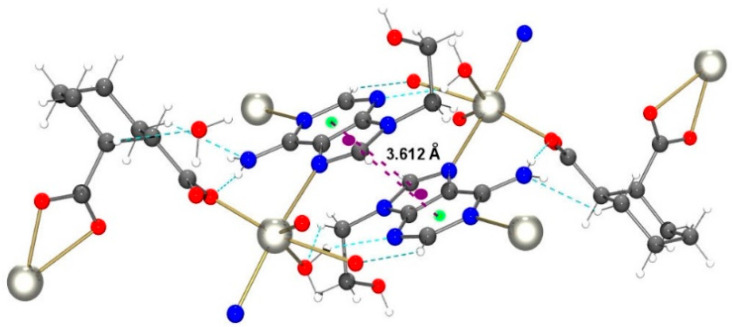
Anti-parallel π-stacking between adenine-9heade moieties in the crystal of the polymer {Cd(µ_2_-({Cd(µ_2_-(cis-1,2-CHDA) µ_2_-(N1,N7-9heade)(H_2_O)]·4H_2_O}_n_.

To emphasize the relevance of the metal chelate-nucleoside molecular recognition in compound **1** reported here, it is worth comparing our results to some structural aspects concerning the polymer {Cd(µ_2_-(cis-1,2-chdc)(µ_2_-N1,N7-9heade) (H_2_O)]·4H_2_O}_n_ (cis-1,2-H_2_chdca is the cis-cyclohexane-1,2-dicarboxylicacid, and cis-1,2-chdc its divalent anion) [15]. In this Cd(II) compound, the main driver causing its polymerization seems to be the utilization of the *cis*-1,2-chdc isomer. Working with the deposited cif file (ANEHOS) corresponding to this polymer in the CSD, we also observed two other relevant aspects. First, cooperation between the coordination bonds Cd-N1(2.364(6) Å) and Cd-N7(2.425(6) Å) with the corresponding two interligand H-bonding interactions (9heade)N6-H···O(coordinated carboxyl, chdc) (2.843 Å, 175.06° or 2.728 Å, 171.23°, respectively) (Figure 10). Second, there was an anti-parallel π-staking interaction between pairs of five- and six-membered rings of neighboring 9heade-adenine moieties, with inter-centroid ring distances of 3.61 Å (Figure 11).

In clear contrast, as mentioned above, π-stacking interactions were missing in compound **1**. Moreover, all attempts to obtain a compound of the type [Cu_2_(µ-EDTA)(µ-N1,N7-9heade)] were unsuccessful. These reactions yielded two distinct kinds of blue crystals, unambiguously identified by single crystal diffraction as the binary polymer {[Cu_2_(µ_4_-EDTA)(H_2_O)_2_]·2H_2_O}_n_ and as compound **1**. As mentioned before, working with 100% excess of 9heade, compound **1** and colorless 9heade were obtained. In our opinion, this emphasizes the suitability of the Cu_2_(µ-EDTA) chelate as a bicephalic receptor of 9heade. In this regard, the molecular nature of the model complex [Cu_2_(µ-EDTA)(9heade)_2_(H_2_O)_2_] in **1** (Figure 2) seems to not be a prerequisite. In fact, closely related compounds of known crystal structures (that not feature H-bonding interligand interactions) have distinct dimensionalities. Indeed, [Cu_2_(μ_2_-EDTA)(py)_2_(H_2_O)_2_]·2H_2_O [23] with pyridine (py) coligand is molecular. However, {[Cu_2_(μ_4_-EDTA)(Him)_2_(H_2_O)_2_]·2H_2_O}_n_ [25] with imidazole or {Cu_2_(μ_4_-EDTA) (3hpy)_2_}_n_ [24] with 3-hydroxypyridine (3hpy) as coligand are polymers.

## 3. Materials and Methods

### 3.1. Synthesis of the Compound [Cu_2_(µ_2_-EDTA)(9heade)_2_(H_2_O)_4_]·3H_2_O (***1***)

This product was obtained by a reaction between stoichiometric amounts of Cu_2_CO_3_(OH)_2_ (green-malachite, Aldrich or blue-azurite, Panreac), H_4_EDTA (Aldrich), and 9heade (TCI) in water. In a typical experiment, Cu_2_CO_3_(OH)_2_ (0.5 mmol, 0.11 g) and H_4_EDTA (0.5 mmol, 0.15 g) were reacted in 100 mL of distilled water, inside a covered Kitasato flask. Its open side outlet allowed for removing CO_2_(the only by-product!) and prevented possible splashes. This reaction mixture was continuously heated (45–50 °C) and stirred for an hour and a half, until a green solid basic copper(II) carbonate was observed. The light blue solution of Cu_2_(μ-EDTA) was allowed to cool to r.t. and was slowly filtered over an Erlenmeyer flask (caution: insufficient reaction times with azurite left black copper(II) oxide on the filter, while unreacted green malachite was observed when used as a copper(II) source). Then, 9heade (0.18 g, 1 mmol) was added to the chelate solution and stirring was continued for half an hour. Mother liquors of the ternary system were filtered again over a crystallization flask, with it being covered with perforated plastic film to control the evaporation of the solvent. Working in this way, the reaction provided the desired product of **1** as flattened parallelepiped crystals (Figure 1). Small amounts of single crystals were manually picked-off from the crystallizer with the aid of a Pasteur pipette, and were allowed to dry on filter paper for crystallographic purposes over 24 h. Furthermore, during the crystallization process, small amounts of the product were time-space removed in the same way, so as to check with FT-IR spectroscopy that only one product was being obtained. Most of the products were collected through filtration, were washed with cool distilled water, and were allowed to air dry in a good yield (ca. 70–80%). Elemental analysis (%): Calc. for C_24_H_44_Cu_2_N_12_O_17_, C 32.04, H 4.93, N 18.68. Found, C 31.96, H, 4.87, N, 18.64. FT-IR [KBr, cm^−1^] (see Supplementary material S1): ν(O-H)_ol_ 3523, ν_as_(NH_2_) 3383, ν_s_(H_2_O) + ν_s_(NH_2_) ~3200, ν_as_(CH_2_) 2981, 2938, δ(H_2_O) + δ(NH_2_) + ν_as_(COO) ~1600, ν_s_(COO) 1388, ν(C-O)_ol_. UV-vis (diffuse reflectance) spectrum (supplementary materials S2): asymmetric d-d band, λ_max_ 734 nm (λ at the intensity barycenter ~800 nm).

### 3.2. Crystal Structure Determination

A crystal of [Cu_2_(µ_2_-EDTA)(9heade)_2_(H_2_O)_4_]·3H_2_O (**1**) was mounted on a glass fiber and used for data collection. The crystal data were collected at 293(2) K, using a Bruker D8 Venture diffractometer (Billerica, MA, USA). Graphite monochromated Cu K(α) radiation (λ = 1.54184 Å) was used throughout. The data were processed with APEX3 [31] and were corrected for absorption using SADABS (transmissions factors: 1.000–0.610) [32]. The structure was solved by direct methods using the program SHELXS-2013 [33], and was refined by full-matrix least-squares techniques against F^2^ using SHELXL-2013 [33]. The positional and anisotropic atomic displacement parameters were refined for all non-hydrogen atoms. Hydrogen atoms were located in difference maps and were included as fixed contributions riding on attached atoms with isotropic thermal parameters 1.2 times those of their carrier atoms. The criteria for a satisfactory complete analysis were the displacement ratios from the root mean square shifts to the standard deviation of less than 0.001, and no significant features were seen in the final difference maps. Atomic scattering factors were taken from the International Tables for Crystallography [34]. Molecular graphics were plotted by DIAMOND [35]. A summary of the crystal data, experimental details and structure refinement (supplementary materials S3), coordination bond lengths and angles (supplementary materials S4), and H-bonding interactions (supplementary materials S5) are provided as Supporting Materials. Crystallographic data for **1** have been deposited in the Cambridge Crystallographic Data Centre, with the reference number 2071137. Copies of this information could be obtained free of charge upon application at http://www.ccdc.cam.ac.uk/products/csd/request (accessed on 17 March 2021).

### 3.3. Computational Methods

Calculations of the non-covalent interactions and molecular electrostatic potential (MEP) surfaces were carried out using the Gaussian-16 [36] and the PBE0-D3/def2-TZVP levels of theory. The D3 dispersion correction proposed by Grimme et al. was used in the calculations [37]. To characterize the interactions in the solid state, the crystallographic coordinates of non-hydrogen atoms were used, and only the position of the H-bonds were optimized. This procedure and level of theory has been successfully used to evaluate similar interactions [38]. The interaction energies were computed by calculating the difference between the energies of the isolated monomers and their assembly. The QTAIM calculations [39] were performed at the same level of theory by means of the AIMAll program [40].

### 3.4. Other Physical Measurements

The elemental analysis was performed with a Thermo Scientific Flash 2000 (Thermo Fisher Scientific Inc, Waltham, MA, USA). Infrared spectra (samples in KBr pellets) were recorded using a Jasco FT-IR 6300 spectrometer (Jasco Analítica, Madrid, Spain). Electronic (diffuse reflectance) spectra were obtained in a Varian Cary-5E spectrophotometer (Agilent Scientific Instruments, Santa Clara, CA, USA) from a grinded crystalline sample. Thermogravimetric analyses (TGA) was carried out (10 °C/min) under air-dry flow (100 mL/min) with a thermobalance Mettler-Toledo TGA/DSC1 (Mettler-Toledo, Columbus, OH, USA), and a series of 49 time-spaced FT-IR spectra were recorded to identify evolved gasses throughout the experiment, using a coupled FT-IR Nicolet 550 spectrometer (Thermo Fisher Scientific Inc., Waltham, MA, USA). X-band EPR measurements were registered on a Bruker ELEXSYS 500 spectrometer (Bruker Analytische Messtechnik GmbH, Karlsruhe, Germany) equipped with a super-high-Q resonator ER-4123-SHQ. For the Q-band studies, EPR spectra were recorded on a Bruker EMX system equipped with an ER-510-QT resonator (Bruker Analytische Messtechnik GmbH, Karlsruhe, Germany). An NMR probe calibrated the magnetic field and the frequency inside the cavity was determined with a Hewlett-Packard 5352B microwave frequency counter. Computer simulations were made with WINEPR-SimFonia, version 1.5. Temperature dependent magnetic measurements were performed between 2 and 300 K, with an applied field of 0.1 T, using a MPMS3 SQUID magnetometer (Quantum Design GmbH, Darmstadt, Germany). The experimental susceptibilities were corrected for the diamagnetism of the constituent atoms by using Pascal tables.

## 4. Concluding Remarks

In this work, we developed a metal chelate suitable to recognize up to two molecules of a synthetic adenine nucleoside. The bicephalic receptor role of dinuclear chelate Cu_2_(µ-EDTA) for two N9-(2-hydroxyethyl) adenine molecules was rigorously supported by the molecular and crystal structure of the designed compound on the basis of previous works and well-known coordination concepts. The efficient metal chelate-nucleoside molecular recognition was featured with the cooperative action of the Cu-N7(9heade) coordination bond and a (9heade)N6-H···O(carboxyl, EDTA) interligand interaction, in good agreement to our theoretical calculations. In addition, the spectral, thermal, and magnetic properties of the novel compound were related to its structural singularities.

## Figures and Tables

**Table 1:** Bond distances (Å) and trans-angles to the six-coordinations of Cu(II) in compound **1**.

Atoms	Distance/Angle
Cu(1)-O(21)	1.943(4)
Cu(1)-O (11)	1.978(4)
Cu(1)-N(7)	2.032(6)
Cu(1)-N(10)	2.039(5)
Cu(1)-O(1)	2.331(5)
Cu(1)-O(2)	2.816(6)
O(21)-Cu(1)-O(11)	164.4(2)
N(7)-Cu(1)-N(10)	174.8(2)
O(1)-Cu(1)-O(2)	163.9(2)

## Supplementary Materials

The following are available online at https://www.mdpi.com/article/10.3390/ph14050426/s1: Additional information on FT-IR spectra (supplementary material S1) and electronic diffuse reflectance spectrum (supplementary material S2) are given. Tables S3–S5 contain relevant information on structure solution and refinement, coordination bond lengths [Å] and angles [°], and H-bonding interactions in the crystal of **1**. A partial view of the compound showing H-bonding interactions between aqua ligand and O-carboxylate acceptors is plotted in supplementary material S6. Supplementary material S7 describes the utilization of FT-IR spectroscopy to identify the evolved gases during the TGA analysis, emphasizing the capability of the most distal aqua ligand. Additionally, supplementary material S8 shows the sensitivity of the ESR spectra to the grinding of the samples.
