# Supplementary material for: Dicopper(II)-EDTA Chelate as a Bicephalic Receptor Model for a Synthetic Adenine Nucleoside
Source: Pharmaceuticals (Basel). 2021 May 2;14(5):426. doi: 10.3390/ph14050426 (PMC8147406; doi:10.3390/ph14050426)

Supplementary material
for the Article in *Pharmaceuticals*

The dicopper(II)-EDTA chelate as bicephalic receptor model for a synthetic adenine nucleoside

María Eugenia García-Rubiño, Antonio Matilla-Hernández[2], Antonio Frontera[3], Luis Lezama[4], Juan Niclós-Gutiérrez,[2] Duane Choquesillo-Lazarte[*,5]

Contents

S1. FT-IR spectra of [Cu_2(μ$_2$-EDTA)(9heade)$_2$(H_2O)$_4$]·3H_2O (1). A) Obtained from blue (azurite) $Cu_2CO_3(OH)_2$. B) Obtained from green (malachite) $Cu_2CO_3(OH)_2$.

S2. Vis-UV spectrum of [Cu_2(μ$_2$-EDTA)(9heade)$_2$ (H_2O)$_4$]·3H_2O (1) (ground solid sample) as obtained by diffuse reflectance.

S3. Crystal data and structure refinement for [Cu_2(μ$_2$-EDTA)(9heade)$_2$(H_2O)$_4$]·3H_2O (1).

S4. Copper(II) coordination bond lengths [Å] and angles [°] in the crystal of [Cu_2(μ$_2$-EDTA)(9heade)$_2$(H_2O)$_4$]·3H_2O (1).

S5. Hydrogen bonds in the crystal of [Cu_2(μ$_2$-EDTA)(9heade)$_2$(H_2O)$_4$]·3H_2O (1) [Å,°].

S6. Partial view of the X-ray structure of compound 1 with indication of the OH···O interactions between the H-atoms of distal aqua ligand and the acceptor O-coordinated atoms from carboxylate groups for two adjacent complexes.**S7.** Effects due to the grinding of samples of 1 on the shape of ESR spectra.

S7. Thermogravimetric analysis (TGA) of [Cu_2(μ$_2$-EDTA)(9heade)$_2$(H2O)$_4$]·3H2O (1).

S8. Effects due to the grinding of samples of 1 on the shape of ESR spectra of 1.

S1. FT-IR spectra of [Cu_2(μ_2-EDTA)(9heade)$_2$(H_2O)$_4$]·3H_2O (1). A) Obtained from blue (azurite) $Cu_2CO_3(OH)_2$. B) Obtained from green (malachite) $Cu_2CO_3(OH)_2$.

A)

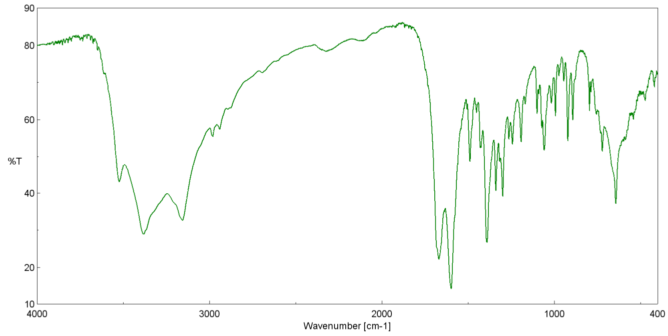

B)

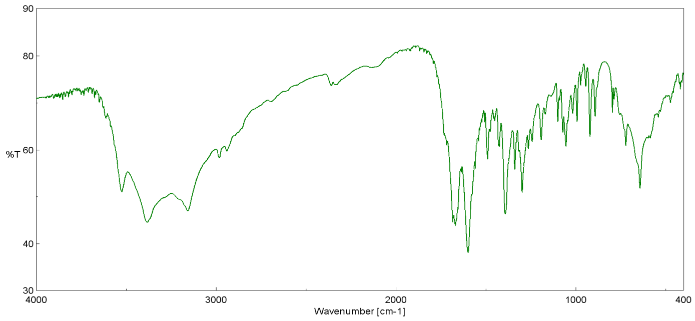

Comment: Small differences between these spectra are related to the partial water lost when the compound (1 mg) is ground with dry KBr (100 mg, Urvasol Merck)

S2. Vis-UV spectrum of [Cu_2(μ_2-EDTA)(9heade)$_2$ (H_2O)$_4$]·3H_2O (1) (ground solid sample) as obtained by diffuse reflectance.

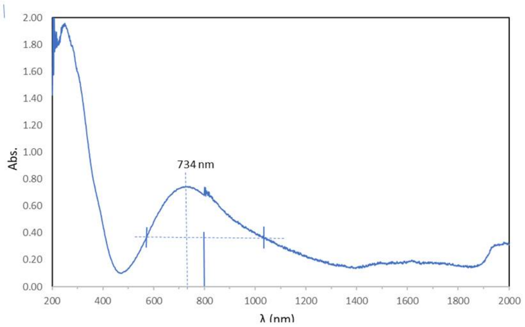

Comment: This spectrum seems not sensitive to the ground of the sample because is the partial loss of the most distal aqua ligand has little impact on the electronic levels of the Cu(II) center in compound 1.

S3. Crystal data and structure refinement for $[Cu_2(\mu_2\text{-EDTA})(9heade)_2(H_2O)_4]\cdot 3H_2O$ (1).

Identification code	17jnac925
Empirical formula	$C_{24}H_{44}Cu_2N_{12}O_{17}$
Formula weight	899.79
Temperature	298(2) K
Wavelength	1.54184 Å
Crystal system, space group	Orthorhombic, Pnma
Unit cell dimensions	a = 9.4875(7) Å, α = 90°<br>b = 37.736(3) Å, β = 90°<br>c = 10.2135(7) Å, γ = 90°
Volume	3656.6(5) $Å^3$
Z, Calculated density	4, 1.634 Mg/m^3
Absorption coefficient	2.228 mm^{-1}
F(000)	1864
Crystal size	0.120 x 0.120 x 0.060 mm
Theta range for data collection	2.342 to 66.634°.
Limiting indices	-11<h<10, -44<k<=44, -12<l<12
Reflections collected / unique	33268 / 3281 [R(int) = 0.1213]
Completeness to θ = 67.684	97.3 %
Absorption correction	Semi-empirical from equivalents
Max. and min. transmission	1.000 and 0.884
Refinement method	Full-matrix least-squares on F^2
Data / restraints / parameters	3281 / 0 / 254
Goodness-of-fit on F^2	1.066
Final R indices [I>2σ(I)]	R1 = 0.0764, wR2 = 0.2034
R indices (all data)	R1 = 0.1177, wR2 = 0.2364
Extinction coefficient	0.00067(13)
Largest diff. peak and hole	1.134 and -0.539 e. $Å^{-3}$

S4. Copper(II) coordination bond lengths [Å] and angles [°] in the crystal of $[Cu_2(\mu_2\text{-EDTA})(9heade)_2(H_2O)_4]\cdot 3H_2O$ (1).

Cu(1)-O(21)	1.943(4)
Cu(1)-O(11)	1.978(4)
Cu(1)-N(7)	2.032(6)
Cu(1)-N(10)	2.039(5)
Cu(1)-O(1)	2.331(5)
Cu(1)-O(2)	2.816(6)
O(21)-Cu(1)-O(11)	164.4(2)
O(21)-Cu(1)-N(7)	97.4(2)
O(11)-Cu(1)-N(7)	94.0(2)
O(21)-Cu(1)-N(10)	83.4(2)
O(11)-Cu(1)-N(10)	84.33(19)
N(7)-Cu(1)-N(10)	174.8(2)
O(21)-Cu(1)-O(1)	103.51(19)
O(11)-Cu(1)-O(1)	87.69(18)
N(7)-Cu(1)-O(1)	86.5(2)
N(10)-Cu(1)-O(1)	98.33(19)
O(21)-Cu(1)-O(2)	92.10(19)
O(11)-Cu(1)-O(2)	77.75(18)
N(7)-Cu(1)-O(2)	87.8(2)
N(10)-Cu(1)-O(2)	87.04(19)
O(1)-Cu(1)-O(2)	163.94(16)

S5. Hydrogen bonds in the crystal of $[Cu_2(\mu_2\text{-EDTA})(9heade)_2(H_2O)_4]\cdot 3H_2O$ (1) [Å, °].

D-H...A	d(D-H)	d(H...A)	d(D...A)	<(DHA)
O(1)-H(1A)...O(22)#2	0.92	1.77	2.675(6)	166.0
O(1)-H(1B)...O(12)#3	0.91	1.90	2.800(7)	168.2
O(2)-H(2A)...O(11)#4	0.93	2.10	2.994(7)	159.3
O(2)-H(2B)...N(1)#5	0.91	2.09	2.853(8)	141.6
O(93)-H(93)...O(3)#3	0.82	1.97	2.756(10)	159.5
N(6)-H(6A)...O(12)#6	0.86	2.20	2.999(7)	155.5
N(6)-H(6B)...O(21)	0.86	1.94	2.790(8)	168.0
O(3)-H(3A)...O(4)#4	1.00	1.73	2.687(14)	158.9
O(3)-H(3B)...O(5)	1.00	1.79	2.762(16)	162.2
O(4)-H(4A)...N(3)	1.00	2.03	2.913(8)	145.4
O(5)-H(5A)...O(93)	0.99	2.00	2.923(14)	154.6

Symmetry transformations used to generate equivalent atoms:
#1 -x-1,-y,-z+1 #2 x+1/2,y,-z+3/2 #3 x+1/2,y,-z+1/2
#4 x-1/2,y,-z+1/2 #5 x-1/2,y,-z+3/2 #6 x,y,z+1
#7 -x-3/2,-y,z-1/2

S6. Partial view of the X-ray structure of compound 1 with indication of the O–H···O interactions between the H-atoms of distal aqua ligand and the acceptor O-coordinated atoms from carboxylate groups for two adjacent complexes.

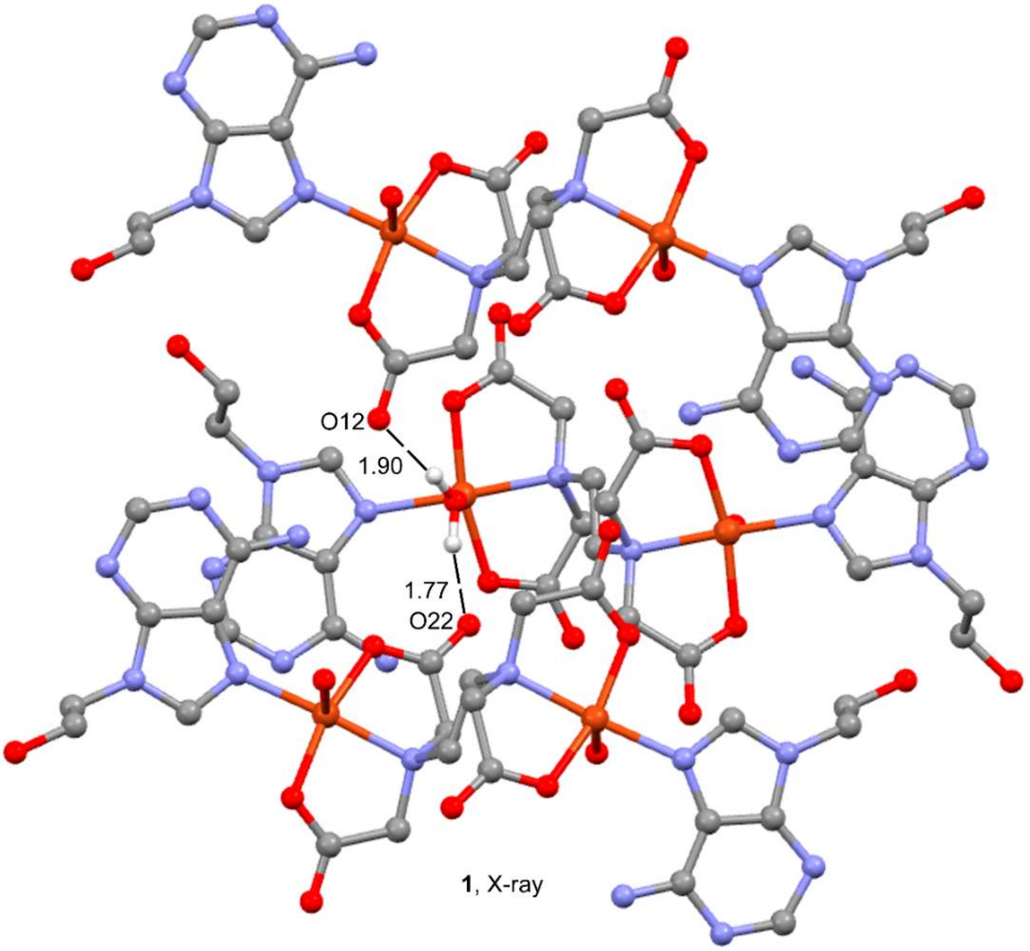

1, X-ray

S7. Thermogravimetric analysis (TGA) of $[Cu_2(\mu_2\text{-}EDTA)(9heade)_2(H_2O)_4]\cdot 3H_2O$ (1).

A) Symmary of results and its asignations.

Compound **1**	$[Cu_2(\mu_2\text{-}EDTA)(9heade)_2(H_2O)_4]\cdot 3H_2O$	$C_{24}H_{44}Cu_2N_{12}O_{17}$	MW 899.77	% H_2O 10.015
Starting TGA formula	$[Cu_2(\mu\text{-}EDTA)(9heade)_2(H_2O)_{3.6}]$ or $Cu_2(\mu\text{-}EDTA)(9heade)_2\cdot 3.6\ H_2O$	$C_{24}H_{30}Cu_2N_{12}O_{10}\cdot 3.6\ H_2O$	MW 838.52	% H_2O 7.735

Step	˚C	minutes	Weight loss (%)	Calculated loss (%)	Gases or final residue
1	20-155	0-13	7.687	7.735	**3.6** H_2O
2	155-270	13-25	19.235	-	H_2O, CO_2
3	270-435	25-42	31.993	-	H_2O, CO_2, CO, N_2O
4	435-595	24-60	21.240	-	H_2O, CO_2(t)*, CO, NH3, N_2O, NO, NO2, CH4(t)*
Residue at	595	60	19.824	18.973	CuO (non pure)

* Trace amounts.

Comment: The starting material (r.t.) have a water content that averagely corresponds to a previous loss of all three crystallization water (3 H_2O) plus a partial (0.4 H_2O) of the most distal aqua ligand. This assumption yield goods agreements between experimental and calculated values for the water loss (first step) and the final residue of CuO at about 600°C.

B) Collection of FT-IR spectra used to identify the evolved gases during the TGA of compound 1.

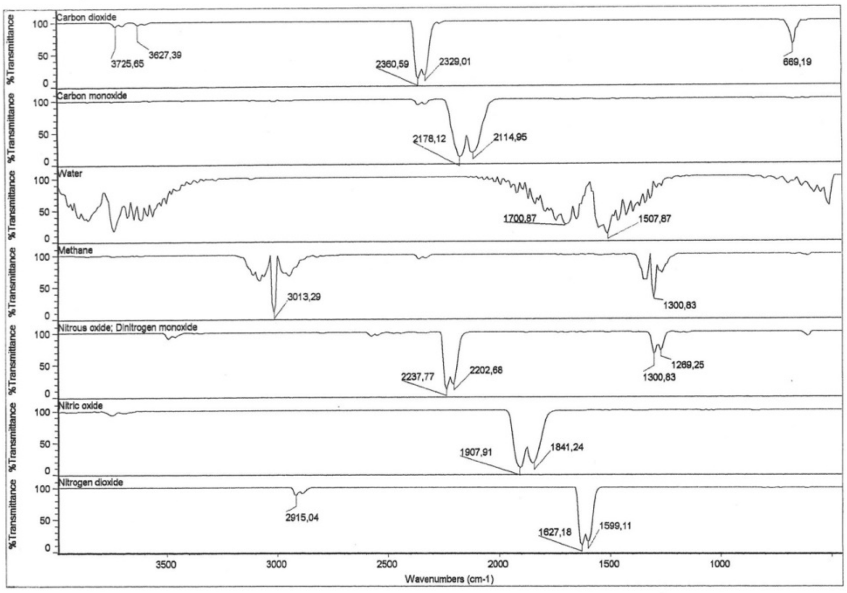

C) Six sequenced and handily-worked sheets showing 42 time-spaced FT-IR spectra to the identification of the evolved gases during the TGA of compound **1**. Next to the right margin the stage (1 to 4) to which they correspond is indicated. Over 60 min., FT-IR spectra shows only retained gases during the experiment.

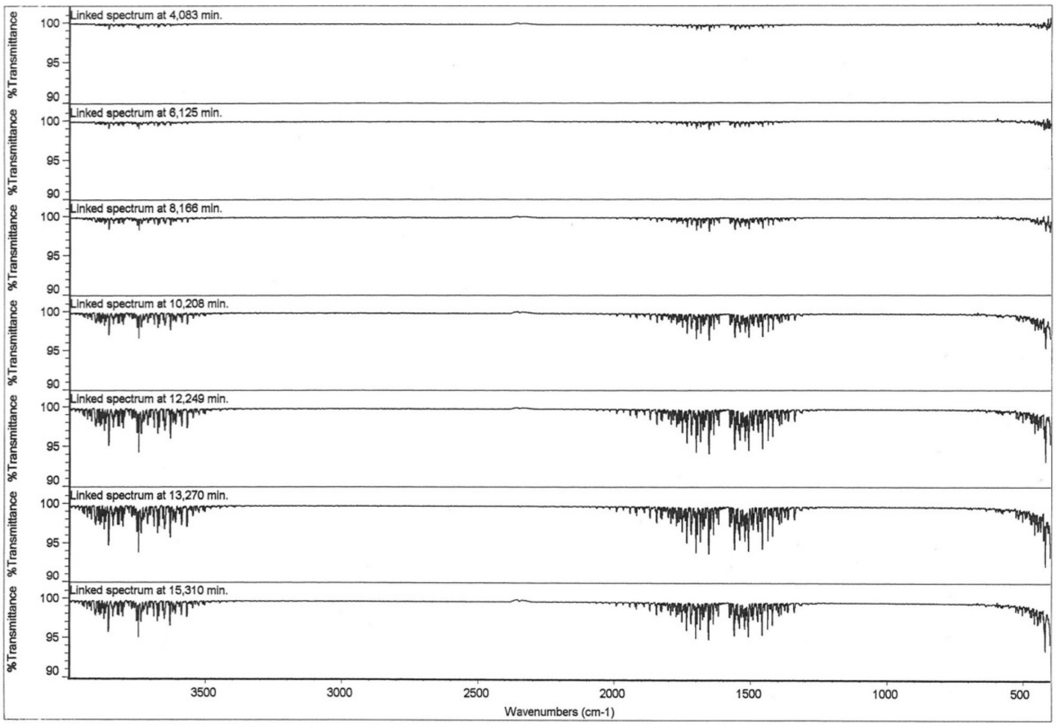

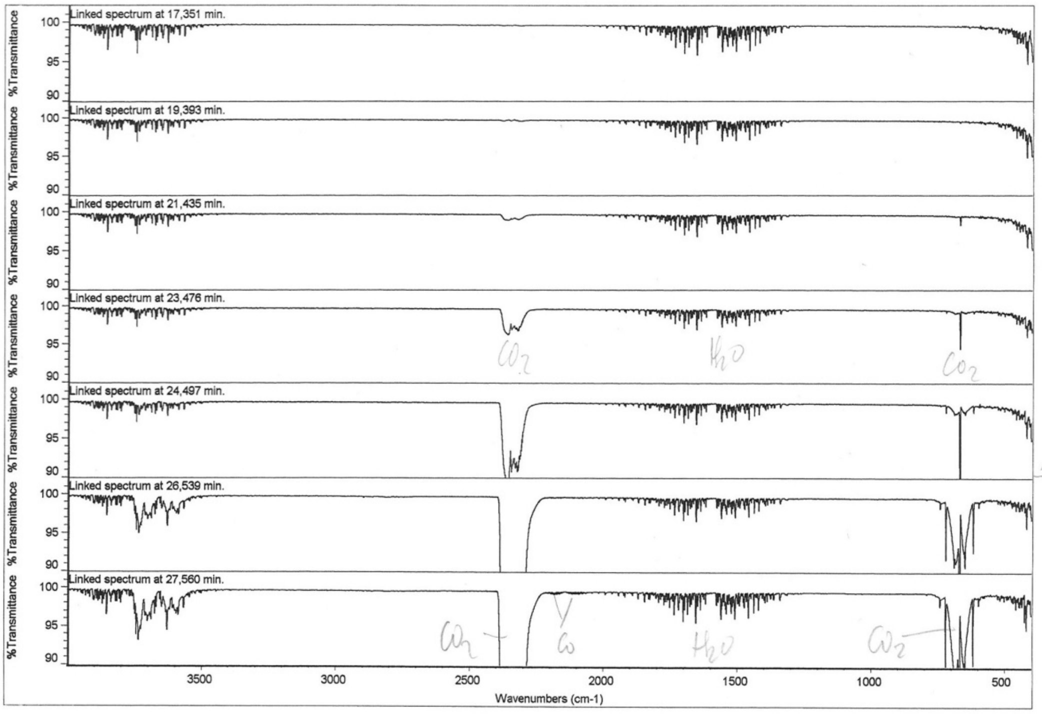

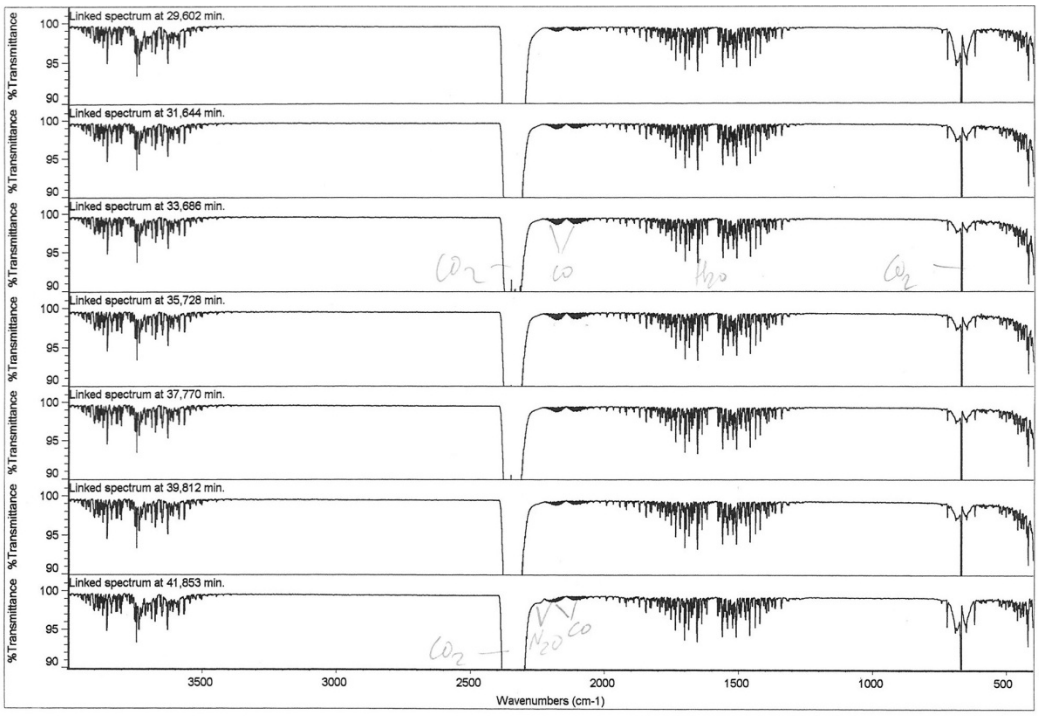
Linked spectrum at 29,602 min.
Linked spectrum at 31,644 min.
Linked spectrum at 33,686 min.
Linked spectrum at 35,728 min.
Linked spectrum at 37,770 min.
Linked spectrum at 39,812 min.
Linked spectrum at 41,853 min.
%Transmittance
100
95
90
3500
3000
2500
2000
1500
1000
500
Wavenumbers (cm-1)
CO_2
CO
H_2O
CO_2
CO_2
N_2O
CO

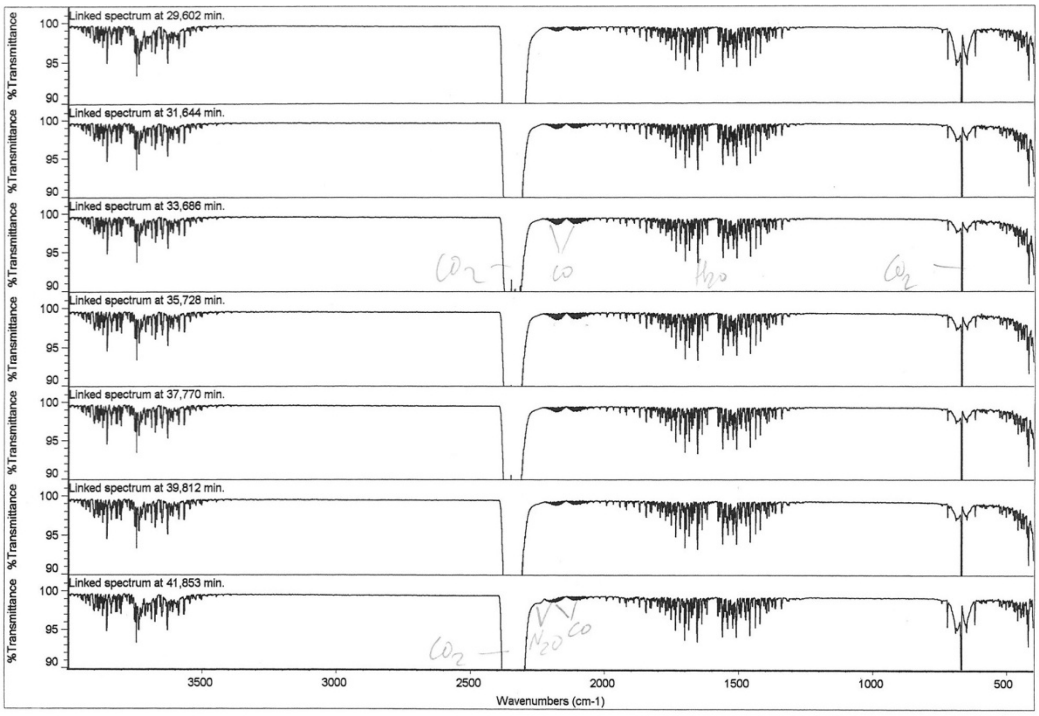
Linked spectrum at 29,602 min.
Linked spectrum at 31,644 min.
Linked spectrum at 33,686 min.
Linked spectrum at 35,728 min.
Linked spectrum at 37,770 min.
Linked spectrum at 39,812 min.
Linked spectrum at 41,853 min.
%Transmittance
100
95
90
3500
3000
2500
2000
1500
1000
500
Wavenumbers (cm-1)
CO_2
CO
H_2O
CO_2
CO_2
N_2O
CO

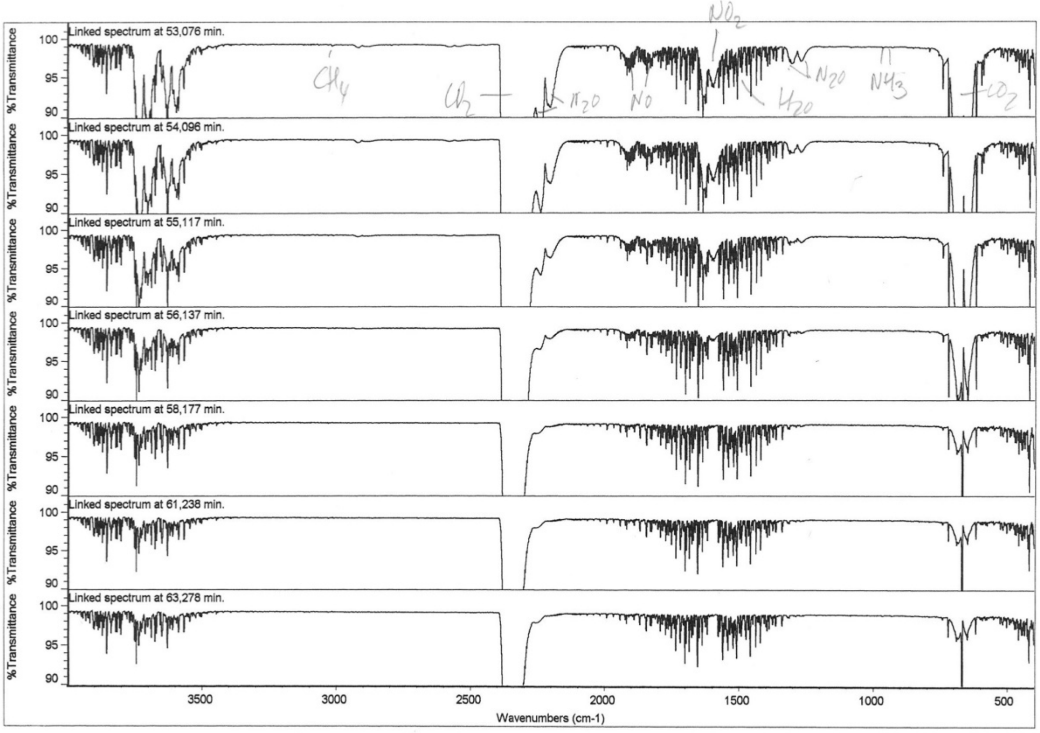
Linked spectrum at 53,076 min.
Linked spectrum at 54,096 min.
Linked spectrum at 55,117 min.
Linked spectrum at 56,137 min.
Linked spectrum at 58,177 min.
Linked spectrum at 61,238 min.
Linked spectrum at 63,278 min.
%Transmittance
100
95
90
3500
3000
2500
2000
1500
1000
500
Wavenumbers (cm-1)
NO2
CH4
CO2
N2O
NO
H2O
N2O
NH3
CO2

11

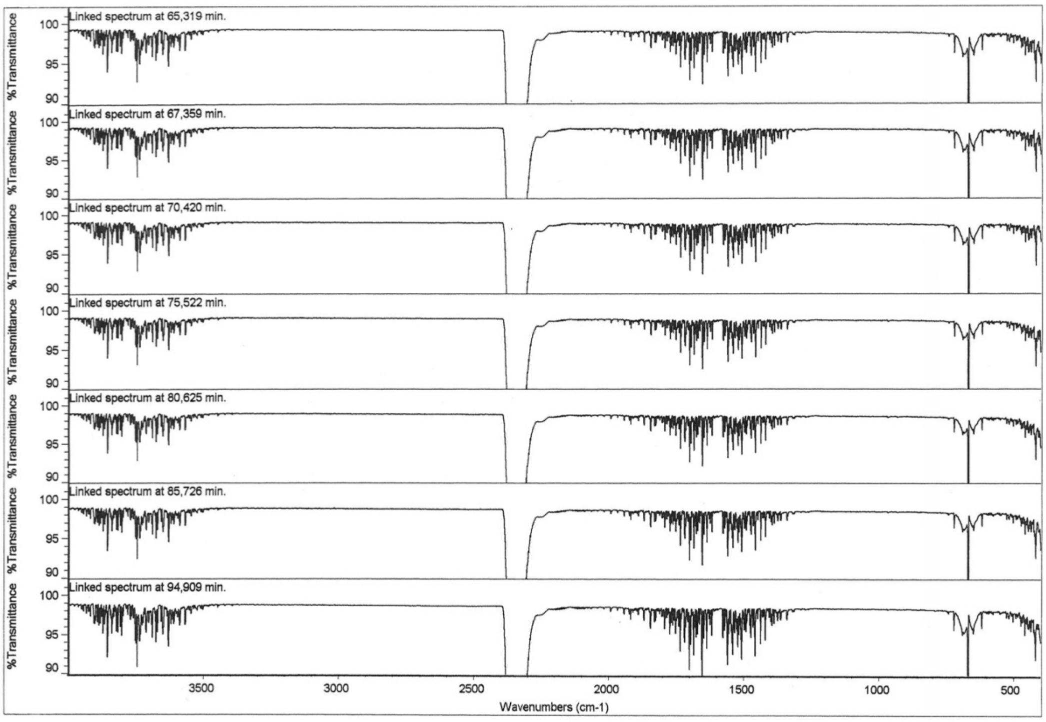
Linked spectrum at 65,319 min.
Linked spectrum at 67,359 min.
Linked spectrum at 70,420 min.
Linked spectrum at 75,522 min.
Linked spectrum at 80,625 min.
Linked spectrum at 85,726 min.
Linked spectrum at 94,909 min.
%Transmittance
100
95
90
3500
3000
2500
2000
1500
1000
500
Wavenumbers (cm-1)

12

S8. Effects due to the grinding of samples of 1 on the shape of ESR spectra of 1.

Comment: The extreme lability of the most distal aqua ligand in the Cu(II) surrounding was marginally observed in the shape of ESR spectra, depending of the grounding of samples of compound 1.

A) ESR spectra on X-band (black, grounded sample; cyan, not grounded sample)

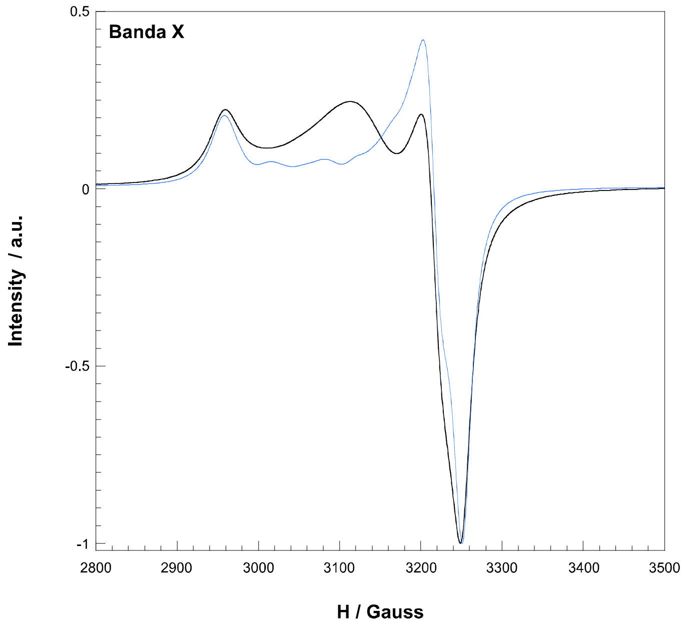

B) ESR spectra on Q-band (red, grounded sample; cyan, smoothly grounded sample)

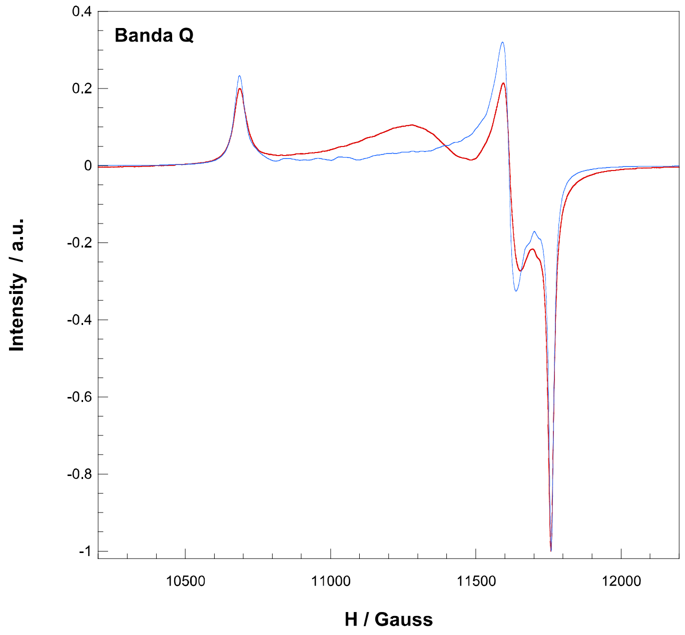

Supplement: Supplementary file 1 [file pharmaceuticals-14-00426-s001.zip › pharmaceuticals-1200831-supplementary.pdf]
